# High HIV and syphilis prevalence among female sex workers in Juba, South Sudan

**DOI:** 10.1371/journal.pone.0239543

**Published:** 2020-09-28

**Authors:** Avi J. Hakim, Alex Bolo, Margaret Werner, Victoria Achut, Joel Katoro, Golda Caesar, Richard Lako, Acaga Ismail Taban, Jennifer Wesson, Alfred G. Okiria

**Affiliations:** 1 Division of Global HIV and TB, US Centers for Disease Control and Prevention, Atlanta, GA, United States of America; 2 South Sudan Ministry of Health, Juba, South Sudan; 3 IntraHealth International, Chapel Hill, NC, United States of America; Rutgers The State University of New Jersey, UNITED STATES

## Abstract

HIV prevalence is estimated to be 2.7% in South Sudan; however, little is known about the young country’s epidemic. We conducted a respondent-driven sampling biobehavioral survey in Juba of female sex workers (FSW) aged ≥15 years who sold or exchanged sex in the last 6 months to learn more about this population. We enrolled 838 FSW from November 2015 to March 2016 and estimated HIV prevalence to be 37.8%. Prevalence of active syphilis was 7.3%. FSW were from South Sudan and most neighboring countries. Comprehensive knowledge of HIV was 11.1% and 64.2% of FSW had never spoken with an outreach worker. In multivariable analysis, HIV was associated with being from Uganda (aOR: 3.3, 95% CI: 1.7–6.1) or Kenya (aOR: 4.3, 95% CI: 1.5–13.0) versus from South Sudan. Our survey suggests that FSW may play a critical role in South Sudan’s HIV epidemic and highlights the importance of tailoring services to the unique needs of FSW of all nationalities in Juba.

## Introduction

HIV has long been a public health concern in South Sudan; however, given the recurrent conflict and humanitarian situation, attention has often been focused on emergency health needs, nutrition and shelter for the displaced. One of the poorest countries in the world, HIV testing and treatment services are extremely limited in South Sudan, with antiretroviral therapy (ART) coverage at 10%, and little is known about HIV in the country [1]. Much of what is known about the epidemic comes from routine HIV program data, antenatal clinic (ANC) surveillance, and UNAIDS Spectrum estimates [2]. HIV prevalence in South Sudan is estimated at 2.7% among 15–49 year olds and there are an estimated 200,000 people living with HIV in the country [1]. ANC sentinel surveillance data suggest that prevalence is highest in the three former Greater Equatoria states in the south of the country [3]. While in other sub-Saharan countries HIV incidence is stabilizing or decreasing and HIV mortality decreasing, the number of both new infections and AIDS-related deaths in South Sudan are estimated to have increased by 3% since 2010 [1]. Though geographically larger than Kenya, South Sudan had only 62 HIV treatment facilities in in 2019 compared to over 2,500 in Kenya.

South Sudan had a surge in sex work following the 2005 signing of the Comprehensive Peace Agreement [4]. Mapping exercises of female sex workers (FSW) in 2010 and 2012 conservatively estimated between 2,000 and 2,500 FSW in Juba, respectively [4, 5]. By 2015–2016, more robust methods produced an estimate of 5,800 FSW in Juba [6]. Sex work is illegal in South Sudan. Sex workers face arrest and harassment from police and other security forces, as well as violence from their clients [4, 7].

At 11.8%, HIV prevalence among FSW is higher than among general population females globally [8]. The odds of becoming HIV infected is 13.5 times greater for FSW than for other women in low and middle income settings [8]. Sex workers are at heightened risk of HIV infection due to the number of sexual partners they have and their frequency of condomless sex [9]. The high prevalence of sexually transmitted infections (STI) among FSW also increases their risk of both acquiring and transmitting HIV [10, 11]. Factors such as stigma, harassment, violence, marginalization, and limited social support, combined with lower education level, poverty, and mobility may increase HIV acquisition and reduce HIV service uptake in FSW [12–14]. Given the relatively low HIV prevalence of South Sudan, sex work likely plays a critical role in the country’s HIV epidemic [15, 16].

To better understand the role of FSW in South Sudan’s HIV epidemic, the South Sudan Ministry of Health (MOH) undertook a biobehavioral survey (BBS) among FSW called The Eagle Survey. Here we describe the FSW population in Juba, South Sudan, their uptake of HIV prevention services, and correlates of HIV infection.

## Methods

### Formative assessment

We conducted a formative assessment in Juba in 2014 to understand FSW social networks and willingness to participate in a BBS. While FSW were largely venue-based, that is they congregated in physical spaces, they had social connections across spaces, and safety considerations for FSW and survey staff at these spaces during data collection led to the selection of respondent-driven sampling (RDS) for recruitment [7]. RDS is a variant of snowball sampling used to produce sampling weights and approximate a random sample[17–19]. The assessment revealed that FSW came from all neighboring countries and spoke multiple languages.

### Study population, setting, and design

The Eagle Survey used RDS to recruit FSW in Juba, South Sudan from November 2015 to March 2016. Eligibility criteria were: female, age ≥15 years, spoke English, Juba Arabic, or Kiswahili, received money, goods, or services in exchange for sex in the past six months, and resided, worked or socialized in Juba for at least the last one month.

### Recruitment and data collection

Four initial seeds were selected based on age, neighborhood, nationality, and influence among peers. Five additional seeds were added later to reach populations underrepresented in the sample compared to formative assessment findings [7]. After eligibility screening and providing verbal informed consent, participants underwent a face-to-face computer-assisted personal interview (Open Data Kit, Washington, US). Interview domains included demographics, social cohesion, stigma, HIV knowledge, sexual history, uptake of HIV and STI services, violence, and history of STI. The two-item Patient Health Questionnaire (PHQ-2) was used to screen for depression and the three-question Alcohol Use Disorders Identification Test for alcohol disorders [20, 21]. Comprehensive knowledge of HIV utilized the UNAIDS definition of correctly answering three questions and rejecting two myths regarding HIV [22]. Participants were also asked, “When you seek health care, do you feel you need to hide that you sell sex or exchange sex for it?” The question asking participants if they ‘smoke or dry out’ their vagina utilized local terminology for this activity.

Upon completion of the interview, participants received HIV pretest counselling. Those who consented to HIV testing were tested by trained staff according to the national testing algorithm of Determine HIV-1/2 (Alere, MA, USA) followed by Uni-Gold (Trinity Biotech, Ireland) for confirmation. Bioline (Standard Diagnostics, South Korea) was used as a tie-breaker. Participants with HIV received CD4 enumeration with the PIMA analyzer (Alere, MA, USA). Quality control of HIV-positive specimens was performed with Geenius HIV-1/2 (Bio-Rad, CA) at the South Sudan National Public Health Lab.

Syphilis testing was conducted using Bioline syphilis 3.0 followed by the Rapid Plasma Reagin (RPR) test. Participants testing positive for HIV were given a referral letter for treatment; those testing positive for syphilis were treated at the study site. Participants were also offered syndromic management of other STI and treatment, as appropriate. Staff were trained to refer all sexually exploited girls under the age of 18 years to partner organizations experienced in providing psychosocial and other protective services to these populations.

Before receiving their test results, participants received compensation for participation (60 South Sudanese Pounds (SSP, approximately $20 USD at the time of data collection), condoms, and three coupons to recruit peers. For the second visit, participants received 20 SSP for transportation and 15 SSP for each successful recruit (approximately $21 USD maximum).

### Data analysis

The primary endpoint for our analysis was HIV infection. Data were analyzed using RDS Analyst version 0.62 (Los Angeles, CA) using Gile’s Successive Sampling Estimator and STATA version 12.0 (College Station, TX). Diagnostics were conducted to assess the sample’s independence from seeds. RDS sampling weights were used in logistic regression to calculate odds ratios (OR) and 95% confidence intervals (95% CI) for bivariate comparisons and variables significant at p<0.1 were included in the weighted multivariable model.

### Ethical approval

Ethical approval for The Eagle Survey was obtained from the South Sudan MOH Ethical Review Board. The protocol was also reviewed in accordance with the US Centers for Disease Control and Prevention (CDC) human research protection procedures and was determined to be research, but CDC investigators did not interact with human subjects or have access to identifiable data or specimens for research purposes.

## Results

Nine seeds were used to recruit and enroll 838 FSW in Juba. The longest chain had 17 waves and convergence was reached on our outcome of interest. Approximately one-fourth (24.4%) of FSW were 15–24 years and 50.0% were aged 30 years or older (Table 1). Ugandans comprised 39.8% of FSW, Congolese 20.7%, and South Sudanese 34.4%. Educational attainment was low, 66.6% of FSW could neither read nor write and 80.3% never reached secondary school. One quarter (25.6%) of FSW had never married and sex work was the main source of income for 97.1%. FSW were mobile, with 17.8% traveling outside Juba to sell sex in the last 12 months, and 73.2% slept in the same place most nights. Nearly two in five (38.9%) FSW screened positive for depression, and 32.5% engaged in harmful drinking behavior. Approximately 29.3% dried out or smoked their vagina. One in five (19.5%) FSW had experienced forced sex in their lifetime.

**Table 1:** Background characteristics of female sex workers in Juba, South Sudan, 2015–2016.

Variable	Known HIV+		Status Unknown Before Survey				Total	
	Sample Proportion (n = 202) %(n)	Population Proportion % (95% CI)	HIV+ Self-reported Unaware		HIV-		Sample Proportion (n = 834)	Population Proportion % (95% CI)
			Sample Proportion (n = 131) %(n)	Population Proportion % (95% CI)	Sample Proportion (n = 501) %(n)	Population Proportion % (95% CI)		
Age(years)	n = 202		n = 131		n = 501		n = 834	
Median (IQR)	31(28–35)		30(27–36)		28(23–33)		29(25–34)	
15–19	1.0	0.9(0.0–1.7)	**	**	4.0	5.5(2.2–8.9)	2.6	3.6(1.7–5.5)
20–24	7.4	9.9(3.3–17.0)	12.2	11.3(4.7–17.5)	25.9	23.3(21.0–31.6)	19.9	20.8(17.1–24.5)
25–29	29.7	29.3(21.2–37.5)	31.3	34.4(23.1–46.4)	24.4	22.4(17.9–26.7)	26.5	25.6(22.1–29.1)
30–34	31.2	30.6(22.3–38.8)	21.4	20.1(11.3–28.5)	23.4	23.3(18.3–28.2)	24.8	24.4(20.9–28.0)
35–39	18.8	16.1(9.4–22.4)	22.9	23.9(12.7–35.1)	11.4	11.3(7.9–14.6)	14.9	14.0(11.3–16.8)
40+	11.9	13.2(5.9–20.4)	12.2	10.5(3.8–16.8)	11.0	11.3(7.7–15.0)	11.3	11.6(8.8–14.3)
Country of Birth	n = 202		n = 127		n = 497		n = 826	
South Sudan	4.9	6.2(2.0–10.5)	26.8	30.7(11.0–51.7)	41.1	46.1(26.0–66.7)	29.9	34.4(21.9–46.9)
Uganda	64.4	66.7(30.9–100.0)	35.4	32.2(15.0–48.8)	35.2	31.3(15.5–46.6)	42.5	39.8(29.7–50.0)
DRC*	19.8	17.0(0.0–43.0)	34.6	34.0(3.8–63.6)	20.5	19.4(8.2–30.5)	22.4	20.7(12.3–29.1)
Kenya	10.9	10.1(0.0–47.8)	2.4	2.1(1.7–2.5)	2.8	2.3(0.0–15.0)	4.8	4.4(0.0–13.3)
Other	0.0	0.0	0.8	1.0(0.0–2.4)	0.4	0.9(0.8–1.0)	0.4	0.7(0.0–1.4)
Literacy	n = 202		n = 127		n = 497		n = 826	
Cannot read or write	56.5	61.7(53.3–70.8)	64.6	69.7(62.3–78.1)	63.0	67.8(62.7–73.6)	61.6	66.6(62.6–70.7)
Can read and write	36.6	33.1(24.7–40.9)	28.3	24.9(16.7–32.2)	31.6	26.4(21.4–31.0)	32.3	27.8(24.1–31.5)
Can read only	6.9	5.3(2.1–8.2)	7.1	5.4(4.5–5.9)	5.4	5.7(2.3–8.9)	6.1	5.5(3.6–7.5)
Highest education								
level	n = 202		n = 127		n = 497		n = 826	
None	37.6	42.2(32.8–52.1)	44.9	50.2(41.8–59.9)	47.3	53.6(47.8–59.9)	44.6	50.1(46.7–53.3)
Primary	38.1	36.4(27.9–44.8)	36.2	33.1(23.7–41.7)	30.0	27.2(22.2–32.0)	32.9	30.2(26.5–34.0)
Secondary	22.3	20.4(13.9–26.7)	17.3	14.7(7.7–21.1)	20.9	18.0(13.4–22.3)	20.7	18.5(14.9–22.1)
Higher	2.0	1.0(0.1–1.7)	1.6	2.0(1.6–2.4)	1.8	1.2(0.3–2.1)	1.8	1.3(0.5–2.0)
Current Marital Status	n = 202		n = 127		n = 497		n = 826	
Single, never married	16.8	16.5(10.7–22.2)	23.6	18.5(10.3–25.6)	29.6	30.0(24.6–35.5)	25.5	25.6(22.0–29.1)
Separated/divorced	56.9	53.9(44.5–62.8)	48.8	57.4(48.1–68.5)	53.1	51.9(45.9–57.7)	53.4	52.9(49.8–56.1)
Widowed	19.8	22.3(13.7–31.4)	25.2	22.7(13.3–31.7)	13.3	13.1(8.9–17.3)	16.7	16.5(13.2–19.8)
Married	6.5	7.3(2.5–12.2)	2.4	1.4(0.0–2.7)	4.0	5.0(3.0–7.2)	4.4	5.0(3.2–6.8)
Religion	n = 202		n = 127		n = 497		n = 826	
Catholic	55.0	55.0(45.5–64.8)	37.8	37.5(24.2–50.5)	36.4	35.0(29.8–40.0)	41.2	41.3(37.2–45.6)
Protestant	26.2	24.4(15.9–33.0)	28.3	28.7(15.3–42.1)	44.1	45.5(39.2–52.1)	37.3	39.1(34.7–43.4)
Muslim	6.4	7.1(1.8–12.5)	12.6	15.0(4.6–25.8)	12.3	11.6(7.0–16.1)	10.9	11.2(8.2–14.1)
Pentecostal	3.0	3.6(0.0–7.4)	6.3	9.1(7.7–10.8)	1.4	4.0(1.8–6.2)	2.5	2.5(1.2–3.9)
Orthodox	5.4	3.5(0.0–8.1)	1.6	1.2(0.8–1.4)	1.4	1.3(0.4–2.2)	2.4	1.8(0.9–2.8)
Other	4.0	6.4(2.9–9.5)	13.4	8.6(3.1–13.5)	4.4	2.6(1.1–4.2)	5.7	4.1(2.8–5.3)
Sex work main source of income	n = 202		n = 127		n = 495		n = 824	
Yes	99.5	99.2(97.6–100.0)	96.1	95.3(91.7–98.6)	97.4	96.8(94.5–99.1)	97.7	97.1(95.7–98.5)
Monthly income (SSP)	n = 200		n = 118		n = 482		n = 800	
0–199	3.0	2.9(0.0–6.3)	4.2	4.9(1.2–8.9)	8.9	6.8(4.1–9.3)	6.7	5.5(3.7–7.3)
200–399	13.0	12.0(6.6–17.2)	22.9	23.2(14.8–31.6)	18.5	16.9(12.7–20.8)	17.8	16.7(13.8–19.7)
400–599	11.5	11.6(6.1–17.3)	14.4	14.4(3.1–25.7)	13.7	12.5(9.0–15.8)	13.3	12.4(9.8–14.9)
600–799	19.5	22.1(14.9–29.8)	25.4	27.3(17.3–37.7)	19.9	23.7(18.8–29.1)	20.6	23.6(19.9–27.4)
800+	53.0	51.5(39.3–63.0)	33.1	30.1(17.9–41.7)	39.0	40.1(34.2–46.2)	41.6	41.7(27.1–46.4)
Length of stay in Juba	n = 202		n = 131		n = 501		n = 834	
<1 year	12.4	12.7(7.5–18.0)	6.1	6.5(3.7–9.3)	7.2	8.5(5.2–12.0)	8.3	9.2(6.6–11.9)
1–4 years	53.2	55.2(46.6–64.2)	51.2	53.6(41.6–63.8)	57.1	56.5(50.5–62.2)	56.1	55.6(51.5–59.7)
5–9 years	31.8	29.3(21.2–37.0)	36.6	36.3(24.9–47.9)	22.6	21.5(17.1–25.9)	27.3	25.4(22.1–28.8)
10+ years	2.5	2.8(0.6–5.0)	6.1	4.6(1.2–7.8)	13.2	13.5(9.0–18.0)	8.4	9.7(7.0–12.4)
Traveled outside of Juba to sell sex in last 12 months	n = 202		n = 131		n = 501		n = 834	
Yes	16.8	13.7(8.4–18.4)	17.6	16.1(9.6–22.2)	17.4	19.7(15.5–24.2)	17.3	17.8(14.9–20.8)
Slept same place most nights	n = 201		n = 127		n = 495		n = 823	
Yes	71.1	68.9(60.5–77.0)	70.9	73.8(63.5–84.7)	73.9	75.0(70.3–79.8)	72.7	73.2(70.3–76.1)
Away from home more than one month in the last six months	n = 202		n = 127		n = 494		n = 823	
Yes	28.7	32.4(24.0–41.4)	17.3	13.1(2.2–23.1)	16.0	12.6(8.9–16.0)	19.9.3	17.6(14.3–20.9)
Screened positive for depression	n = 202		n = 125		n = 492		n = 819	
Yes	38.1	36.4(27.9–44.4)	45.6	45.3(35.4–54.7)	41.5	39.0(33.4–44.2)	41.3	38.9(34.7–43.1)
Harmful drinking behavior	n = 202		n = 126		n = 495		n = 823	
Yes	40.1	38.4(29.6–47.1)	32.5	33.5(22.0–45.1)	31.1	29.5(23.5–35.3)	33.5	32.5(28.3–36.6)
Dry or smoke out vagina	n = 202		n = 123		n = 488		n = 813	
Yes	43.6	39.4(30.6–47.6)	26.8	22.7(7.7–36.7)	30.9	26.8(21.3–31.8)	33.5	29.3(25.2–33.3)
Experienced forced sex in lifetime	n = 202		n = 125		n = 492		n = 819	
Yes	18.3	15.5(10.2–20.4)	17.6	19.9(15.5–24.4)	19.5	19.4(11.7–27.2)	18.9	19.5(15.9–22.9)

*DRC: Democratic Republic of Congo.

**Value suppressed because of small size.

Approximately 1 in 5 FSW (21.2%) had sex before age 15 years (Table 2). The median time engaged in sex work was 3 years, with 11.4% of women selling sex for less than one year. Nearly one in three (29.1%) FSW had an agent that helped them meet clients. Just over three-quarters (76.0%) of FSW had more than 20 clients in the last six months. Condom use at last sex with a client was 72.1%. By HIV status, condom use at last sex with a client was 91.6% among FSW who knew they were living with HIV, 75.2% among those with undiagnosed HIV, and 63.8% among those who were HIV-negative. Condom breakage was experienced by 43.9% of FSW in the last six months.

**Table 2:** Sex work characteristics among FSW in Juba, South Sudan 2015–2016.

Variable	Known HIV+		Status Unknown Before Survey				Total	
	Sample Proportion (n = 202)	Population Proportion %(95%CI)	HIV+ Unaware		HIV-		Sample Proportion (n = 834)	Population Proportion % (95% CI)
			Sample Proportion (n = 131)	Population Proportion % (95% CI)	Sample Proportion (n = 501)	Population Proportion % (95% CI)		
Age at sexual debut	n = 201		n = 122		n = 473		n = 796	
Median (IQR)	16(15–17)		15(14–17)		15(15–17)		15(15–17)	
<15	18.9	17.9(9.2–26.5)	33.6	34.4(22.2–46.7)	22.0	19.9(15.6–23.9)	22.6	21.2(17.8–24.6)
15–17	57.7	62.8(53.7–72.7)	45.9	46.9(35.8–58.2)	53.3	56.3(50.9–62.0)	53.4	56.6(52.4–60.7)
18+	23.4	19.4(12.0–25.9)	20.5	18.7(11.2–25.9)	24.7	23.8(19.0–28.7)	24.0	22.3(18.8–25.6)
Age at first exchange sex	n = 199		n = 123		n = 484		n = 806	
Median (IQR)	26(23–31)		26(22–30)		24(20–28)		25(20–29)	
<18	6.5	6.2(2.2–10.1)	4.9	5.1(2.4–7.9)	12.2	13.5(9.2–17.9)	9.7	10.9(8.1–13.7)
18–24	26.1	26.1(18.0–34.0)	34.1	38.6(29.4–48.6)	41.5	41.9(36.7–47.1)	36.6	37.8(33.9–41.7)
25–29	34.7	37.7(30.0–46.1)	29.3	25.9(15.5–35.5)	29.1	26.9(22.0–31.8)	30.5	29.1(25.5–32.8)
30+	32.7	30.0(21.9–37.8)	31.7	30.4(20.3–40.3)	17.2	17.7(13.4–22.0)	23.2	22.3(18.7–25.8)
Time engaged in sex work	n = 199		n = 123		n = 485		n = 807	
Median (IQR)	3(1–4)		3(2–6)		2(1–5)		3(1–5)	
<1 year	15.6	16.9(10.4–23.7)	6.5	6.4(1.5–11.3)	9.7	10.5(6.7–14.5)	10.6	11.4(8.5–14.2)
1–4 years	60.3	59.4(50.7–68.0)	54.5	52.7(41.1–63.7)	64.9	64.0(58.5–69.4)	62.2	61.5(57.4–65.6)
5–9 years	21.1	21.4(14.3–28.4)	31.9	30.6(20.5–40.7)	18.6	18.3(13.8–22.8)	21.1	20.8(17.5–24.2)
10+ years	3.0	2.3(0.1–4.4)	8.1	10.4(4.7–16.6)	6.8	7.2(4.4–9.9)	6.0	6.3(4.4–8.3)
Have agent that helps them meet clients	n = 202		n = 125		n = 493		n = 820	
Yes	20.3	20.0(13.6–26.3)	27.2	28.3(17.8–38.8)	31.6	33.1(27.8–38.5)	28.2	29.1(25.4–32.9)
Number of main male sex partners in last 6 months	n = 193		n = 125		n = 490		n = 808	
0	48.2	48.1(37.2–59.0)	63.2	63.3(53.4–73.2)	56.7	59.1(53.1–65.2)	55.7	56.9(52.4–61.4)
1	40.9	39.1(29.2–48.6)	27.2	23.9(16.2–30.9)	24.5	24.6(19.7–29.8)	28.8	28.2(24.2–32.3)
2+	10.9	12.8(6.8–19.2)	9.6	12.8(5.9–20.3)	18.8	16.3(12.4–19.9)	15.5	14.9(12.1–17.7)
Number of commercial sex partners in last 6 months	n = 110		n = 84		n = 324		n = 518	
1–10	12.7	12.8(5.7–19.8)	9.5	10.8 (2.1–19.9)	17.3	17.0(12.6–21.4)	15.1	15.2(11.8–18.5)
11–20	14.6	13.5(6.3–20.4)	5.9	6.3 (1.6–11.1)	9.9	8.0(4.8–10.9)	10.2	8.8(6.2–11.3)
21–50	32.7	38.0(29.2–47.7)	42.9	40.4 (26.6–53.9)	32.1	32.4(26.2–38.5)	34.0	34.4(29.7–39.2)
51+	40.0	35.8(25.9–45.1)	41.7	42.5 (24.6–60.2)	40.7	42.7(26.9–48.7)	40.7	41.6(36.8–46.4)
Used a condom at last sex with commercial sex partner	n = 196		n = 121		n = 473		n = 790	
Yes	93.4	91.6(85.9–97.0)	79.3	75.2(48.1–100.0)	67.6	63.8(55.5–71.7)	75.8	72.1(66.2–77.9)
Had a condom break during vaginal or anal sex in last 6 months	n = 108		n = 108		n = 405		n = 621	
Yes	75.0	72.6(60.9–83.7)	54.6	51.5(40.5–63.8)	38.0	35.3(28.9–41.4)	47.3	43.9(38.2–49.4)

Only 12.5% of FSW had comprehensive knowledge of HIV (Table 3), and 79.0% thought vaginal sex was the riskiest form of sex (results not shown). Though 27.3% of FSW spoke with a peer educator or outreach worker about HIV in the last three months, 64.2% had never spoken with one. When accessing healthcare services, 16.2% of FSW felt the need to hide that they sell sex from healthcare providers. Approximately three-quarters (78.8%) had ever tested for HIV. HIV prevalence among FSW was 37.8% (95% CI: 33.1–42.5), and 36.8% (95% CI: 29.6–44.0) of those living with HIV were unaware they were infected (results not shown). Nearly two-thirds (65.0%) of FSW living with HIV had a CD4 count less than 500. Prevalence of lifetime and active syphilis infection was 12.0% and 7.3%, respectively.

**Table 3:** Knowledge and healthcare service utilization characteristics among FSW in Juba, South Sudan 2015–2016.

Variable	Known HIV+		Status Unknown Before Survey				Total	
	Sample Proportion (n = 202)	Population Proportion % (95%CI)	HIV+ Unaware		HIV-		Sample Proportion (n = 834)	Population Proportion % (95% CI)
			Sample Proportion (n = 131)	Population Proportion % (95% CI)	Sample Proportion (n = 501)	Population Proportion % (95% CI)		
Comprehensive knowledge of HIV	n = 202		n = 131		n = 501		n = 834	
Yes	11.9	11.4(6.5–16.3)	14.5	12.9(5.8–19.6)	13.4	12.8(9.4–16.2)	13.2	12.5(10.1–14.9)
Last time talked with peer educator or outreach worker:	n = 201		n = 125		n = 491		n = 817	
In the last 30 days	28.9	29.7(21.4–38.2)	11.2	12.1(4.3–20.1)	15.5	12.5(8.4–16.3)	18.1	16.5(13.2–19.8)
Last 1–3 months	20.4	23.5(14.0–33.2)	7.2	8.3(2.7–14.0)	6.7	6.3(3.7–8.8)	10.2	10.8(7.9–13.7)
Last 4–12 months	8.4	7.2(2.9–11.2)	8.0	6.3(2.1–10.0)	7.3	5.9(3.7–8.0)	7.7	6.2(4.5–7.9)
More than a year ago	5.5	5.3(2.2–8.3)	2.4	1.3(0.0–3.9)	1.4	1.2(0.0–2.4)	2.6	2.2(1.2–3.3)
Never	36.8	34.4(25.7–42.8)	71.2	72.0(61.9–82.6)	69.1	74.1(68.9–79.8)	61.4	64.2(59.6–68.9)
Experienced STI symptoms in last 12 months	n = 202		n = 126		n = 492		n = 820	
Yes	34.7	34.1(25.8–42.5)	27.8	27.6(19.1–36.0)	28.9	25.6(21.1–29.7)	30.1	27.8(24.3–31.3)
Felt the need to hide sex work when seeking healthcare	n = 199		n = 123		n = 472		n = 794	
Yes	18.1	16.3(10.7–21.8)	13.0	11.5(5.4–17.4)	16.5	16.9(12.4–21.5)	16.4	16.2(13.1–19.3)
Ever been tested for HIV			n = 126		n = 497		n = 825	
Yes	-	-	64.3	61.5(49.7–72.2)	75.3	74.4(69.2–79.6)	79.6	78.8(75.0–82.4)
CD4 Count	n = 202		n = 131				n = 333	
<500	62.4	62.7(53.5–71.9)	64.1	68.8(59.1–79.4)	-	-	63.1	65.0(58.3–71.9)
500+	37.6	37.3(28.1–46.5)	35.9	31.2(20.6–40.9)			36.9	35.0(28.1–41.7)
Infected with syphilis	n = 202		n = 131		n = 501		n = 834	
Ever	14.4	8.6(4.3–13.0)	18.3	18.7(11.5–26.0)	10.4	10.1(7.1–13.1)	12.6	12.0(6.0–18.0)
Active	8.4	12.6(7.3–17.6)	10.7	8.5(4.1–12.6)	6.8	6.7(4.1–9.2)	7.8	7.3(5.3–9.3)

In multivariable analysis, living with HIV was associated with being from Uganda (aOR: 3.3, 95% CI: 1.7–6.1) or Kenya (aOR: 4.3, 95% CI: 1.5–13.0) versus from South Sudan (Table 4). It is plausible that being from the Democratic Republic of Congo (DRC) is also associated with HIV (aOR: 1.8, 95% CI: 0.9–3.5). Age greater than 15–24 years (25–29 years: aOR: 3.5, 95% CI: 1.8–6.9; 30–34 years: aOR: 2.4, 95% CI: 1.1–5.2; 35+ years: aOR: 2.8, 95% CI: 1.1–7.0) was also associated with having HIV. It is plausible that living in Juba for 5–9 years versus less than one year (aOR: 1.7, 95% CI: 1.0–3.1) and having ever spoken with a peer educator or outreach worker was also associated with HIV (aOR: 1.5, 95% CI: 1.0–2.3).

**Table 4:** Bivariate and multivariate model for HIV infection among FSW in Juba, 2015–2016 (N = 834).

Variable	HIV prevalence	OR (95% CI)	P-value	aOR (95% CI)	P-value
Country of Birth					
South Sudan	16.7	ref	<0.001	Ref	0.070
Uganda	51.3	5.3(3.3–8.3)		3.3(1.7–6.1)	
DRC	42.1	3.6(2.2–6.1)		1.8(0.9–3.5)	
Kenya	65.0	9.3(3.6–24.3)		4.3(1.5–13.0)	
Other	19.1	1.2(0.1–13.9)		0.8(0.1–9.5)	
Age					
15–24	17.3	ref	<0.001	Ref	0.119
25–29	45.8	4.0(2.3–7.1)		3.5(1.8–6.9)	
30–34	41.1	3.3(1.9–5.9)		2.4(1.1–5.2)	
35+	45.6	4.0(2.2–7.1)		2.8(1.1–7.0)	
Highest education level					
None	33.9	ref	0.204		
Primary	44.0	1.5(1.0–2.3)			
Secondary	38.4	1.2(0.8–1.9)			
Higher	40.2	1.3(0.4–4.5)			
Marital status					
Single, never married					
	25.9	ref	0.001	Ref	0.045
Separated/divorced	39.3	1.8(1.2–2.8)		1.0(0.6–1.7)	
Married	38.2	1.8(0.8–4.1)		0.9(0.3–2.4)	
Widowed	51.1	3.0(1.7–5.2)		1.7(0.9–3.1)	
Time in Juba					
<1 year	41.2	ref	<0.001	Ref	0.990
1–4 years	36.2	0.8(0.5–1.3)		0.9(0.5–1.4)	
5–9 years	47.3	1.3(0.8–2.1)		1.7(1.0–3.1)	
10+ years	11.9	0.2(0.1–0.5)		0.5(0.2–1.2)	
Traveled outside Juba					
No	39.3	ref	0.101		
Yes	31.0	0.7(0.5–1.1)			
Dry or smoke out vagina					
No	35.8	ref	0.093	Ref	0.393
Yes	43.4	1.4(0.9–2.0)		1.0(0.6–1.5)	
Sex work main source of income					
No	30.0	ref	0.526		
Yes	38.1	1.4(0.5–4.4)			
Screened positive for depression					
No	37.8	ref	0.884		
Yes	38.4	1.0(0.7–1.5)			
Harmful drinking behavior					
No	35.5	ref	0.085	Ref	0.355
Yes	43.1	1.4(1.0–2.0)		1.1(0.7–1.8)	
Age at sexual debut					
<15	43.1	ref	0.306		
15–17	39.1	0.8(0.5–1.3)			
18+	33.7	0.7(0.4–1.1)			
Age at first exchange sex					
<18	21.0	ref	<0.001	Ref	0.804
18–24	31.1	1.7(0.9–3.4)		1.0(0.4–2.2)	
25–29	43.4	2.9(1.4–5.8)		0.8(0.3–2.0)	
30+	51.3	4.0(1.9–8.2)		1.1(0.4–3.0)	
Time engaged in sex work					
<1 year	43.3	ref	0.177		
1–4 years	35.4	0.7(0.4–1.3)			
5–9 years	45.4	1.1(0.6–2.1)			
10+ years	31.0	0.6(0.2–1.4)			
Have agents that helps FSW meet clients					
No	41.3	ref	0.011	Ref	0.822
Yes	29.9	0.6(0.4–0.9)		0.9(0.6–1.5)	
Number of commercial sex partners in last 6 months					
1–10	26.9	ref	0.331		
11–20	40.6	1.9(0.8–4.5)			
21–50	38.8	1.7(0.9–3.4)			
51+	32.2	1.3(0.7–2.5)			
Used a condom at last sex with commercial sex partner					
No	19.8	ref	<0.001	Ref	0.018
Yes	45.6	3.4(2.1–5.4)		1.7(0.9–3.1)	
Comprehensive knowledge of HIV					
No	38.0	ref	0.742		
Yes	36.1	0.9(0.6–1.5)			
Ever spoken with a peer educator or outreach worker about HIV					
No	28.5	ref	<0.001	Ref	0.002
Yes	53.8	2.9(2.0–4.2)		1.5(1.0–2.3)	
Experienced STI symptoms in last 12 months					
No	36.0	ref	0.113		
Yes	43.2	1.4(0.9–2.0)			
Active syphilis					
No	37.3	ref	0.359		
Yes	43.9	1.3(0.7–2.4)			

Because behaviors may change upon learning one has HIV infection, we also compared HIV-negative FSW to those who were unaware that they were living with HIV (undiagnosed) to understand characteristics associated with HIV infection before any potential behavior change that might result from learning one has HIV. In this analysis we found that compared to HIV-negative women, women with undiagnosed HIV were more likely to be 25–29 years old (aOR: 4.9, 2.0–12.0) (Table 5). Those whose sexual debut was at age 15–17 years, or 18 years or later were less likely to have undiagnosed infection than those who started before age 15 years (aOR 0.5, 95% CI: 0.3–0.9 and aOR 0.4, 95% CI: 0.2–0.9, respectively). It is plausible that those from DRC were more likely than South Sudanese to be undiagnosed (aOR: 1.9, 95% CI: 0.9–4.0).

**Table 5:** Bivariate and multivariate associations of having undiagnosed HIV infection versus being HIV-negative (n = 632).

Variable	OR (95% CI)		aOR (95% CI)	
Country of Birth				
South Sudan	ref	0.047	ref	0.219
Uganda	1.5(0.9–2.7)		1.2(0.6–2.4)	
DRC	2.6(1.4–4.8)		1.9(0.9–4.0)	
Kenya	1.4(0.3–5.6)		1.3(0.3–6.5)	
Other	1.6(0.1–19.0)		0.7(0.1–9.7)	
Age				
15–24	ref	0.001	ref	0.144
25–29	4.3(2.0–9.2)		4.9(2.0–12.0)	
30–34	2.4(1.1–5.5)		2.6(0.8–8.2)	
35+	4.3(2.0–9.2)		2.8(0.8–9.6)	
Highest education level				
None	ref	0.613		
Primary	1.3(0.8–2.2)			
Secondary	0.9(0.4–1.7)			
Higher	1.7(0.3–8.9)			
Marital Status				
Single, never married	ref	0.005	ref	0.152
Separated/Divorced	1.8(1.0–3.2)		1.3(0.7–2.7)	
Married	0.4(0.1–1.7)		0.4(0.1–1.8)	
Widowed	2.8(1.4–5.7)		2.2(0.9–5.2)	
Dry or smoke out vagina				
No	ref			
Yes	0.8(0.5–1.4)	0.421		
Screened positive for depression				
No	ref			
Yes	1.3(0.8–2.1)	0.286		
Harmful drinking behavior				
No	ref			
Yes	1.2(0.7–2.0)	0.478		
Age at sexual debut				
<15	ref	0.016	ref	0.011
15–17	0.5(0.3–0.8)		0.5(0.3–0.9)	
18+	0.5(0.2–0.9)		0.4(0.2–0.9)	
Age at first exchange sex				
<18	ref	0.028	ref	0.991
18–24	2.4(0.9–6.7)		1.6(0.5–4.7)	
25–29	2.5(0.9–7.1)		0.8(0.2–3.0)	
30+	4.5(1.6–13.0)		1.5(0.3–7.1)	
Has agent to help FSW to meet clients				
No	Ref			
Yes	0.8(0.5–1.4)	0.405		
Number of commercial sex partners in last 6 months				
1–10	ref	0.546		
11–20	1.2(0.3–4.9)			
21–50	2.0(0.7–5.1)			
51+	1.6(0.6–4.0)			
Used a condom at last sex with commercial sex partner				
No	ref		ref	
Yes	1.7(1.0–3.0)	0.060	1.7(0.9–3.5)	0.135
Comprehensive knowledge of HIV				
No	ref			
Yes	1.0(0.5–1.9)	0.998		
Ever spoken with a peer educator or outreach worker about HIV				
No	ref			
Yes	1.1(0.6–1.8)	0.786		
Experienced STI symptoms in last 12 months				
No	ref			
Yes	1.1(0.7–1.9)	0.697		
Active syphilis infection				
No	ref			
Yes	1.3(0.6–2.8)	0.491		

## Discussion

This is the first BBS to characterize HIV among FSW in South Sudan, a country about whose HIV epidemic, particularly among FSW, little is known. HIV prevalence among FSW was 10–13 times higher than among general population adult females in Juba [23]. Though most FSW had been in Juba for over one year, mobility was high with 26.8% not sleeping in the same place most nights and nearly one-fifth having sold sex outside the city in the last 12 months. This unstable living situation may contribute to the high prevalence of depression among FSW and engagement in harmful drinking behaviors [24]. Our findings show the importance of ensuring all individuals, regardless of nationality and language ability, have access to HIV prevention, testing, and treatment services in South Sudan. The diverse nationality of sex workers suggests a role for cross-border collaboration by neighboring governments and the need for a regional approach to HIV in South Sudan. The mobility inherent among foreign sex workers and evidenced among FSW in Juba necessitates innovative approaches to ensure HIV-infected FSW are able to stay on treatment when relocating.

Younger girls and women may be especially vulnerable to HIV and sexual exploitation and should be targeted for HIV testing and psychosocial support services. Though nearly half of FSW began selling sex by age 24 years, only 24.4% of FSW in Juba were less than this age, suggesting that women may be starting to sell sex at a later age, that they start selling sex elsewhere and then come to Juba, perhaps to earn more money, or that we did not fully reach into the networks of younger FSW.

Interestingly, condom use at last commercial sex was 91.6% among FSW who knew they were living with HIV, 75.2% among those with undiagnosed HIV, and 63.8% among those who were HIV-negative. This difference could be because FSW who know they have HIV are taking steps to prevent transmitting HIV to clients or prevent transmission or acquisition of other STIs. Social desirability bias is also possible. As there is no significant difference in syphilis prevalence among FSW by HIV status, it is likely that FSW who are aware of their HIV infection may use condoms at a similar rate as other FSW. Their understanding of HIV may have increased after being diagnosed with HIV and they may have reported socially desirable behaviors.

A high proportion of FSW experienced a broken condom in the last six months. This may be because many FSW dry out their vagina and because lubricant is not readily available in Juba. Neither having a condom rupture nor vaginal drying were associated with HIV.

Only one in ten FSW had a comprehensive knowledge of HIV, indicating the continued importance of basic HIV and sexual health education among FSW. Though one-quarter of FSW had spoken with an outreach worker or peer educator in the last three months, nearly two-thirds had never done so, suggesting that outreach efforts may have been reaching the same set of sex workers over and over again. Peer education and outreach efforts should be closely monitored to ensure they continue to reach new individuals. Additionally, given the success of our survey at reaching FSW who had never been reached by outreach workers, social network strategies could be used to reach these women [25–27].

STI services have been found to be more important to FSW than HIV services [28]. With 7.3% of FSW having syphilis and 27.8% experiencing symptoms of an STI in the last 12 months, FSW sexual health needs remain unmet. Similarly, over one-third of FSW living with HIV are unaware that they are infected. Integrated HIV and STI services may prove attractive to FSW in Juba and could open the door to provider-initiated HIV testing.

Our survey is limited by the cross-sectional nature of our sample as well as by our inability to sample many women from subpopulations of Ethiopians or Eritreans due to the exceptional concealment of their engagement in sex work as well as the fact that speaking Amharic was not included in the survey eligibility criteria [7]. There may also be response bias due to the face-to-face nature of our interviews. Using audio-computer-assisted self-interviews could help mitigate this bias but would be challenging with so many languages spoken by FSW [29, 30]. The compensation level was determined through community consultation; it could have had an effect on participation.

Our survey reveals the diversity of FSW in Juba and highlights the importance of tailoring services to the unique needs of FSW of all nationalities in the country. Further research is necessary to understand if women of different nationalities have differential access to services and the reasons for this, including linguistic, cultural, and legal. The high prevalence of HIV and active syphilis among FSW in Juba highlight the urgent need for HIV and STI services targeting FSW. Our findings also highlight the potential impact optimized outreach and case finding approaches could have for FSW and their clients.
